# Methamphetamine Blocks Adenosine A_2A_ Receptor Activation via Sigma 1 and Cannabinoid CB_1_ Receptors

**DOI:** 10.3390/ijms22052743

**Published:** 2021-03-09

**Authors:** Mireia Casanovas, Irene Reyes-Resina, Alejandro Lillo, Jaume Lillo, Raul López-Arnau, Jorge Camarasa, Elena Escubedo, Gemma Navarro, Rafael Franco

**Affiliations:** 1 Biology School, Department of Biochemistry and Molecular Biomedicine, University of Barcelona, 08028 Barcelona, Spain; mireiacasanovas@ub.edu (M.C.); ire-reyes@hotmail.com (I.R.-R.); jaumelillo@ub.edu (J.L.); 2 Centro de Investigación Biomédica en Red Enfermedades Neurodegenerativas (CiberNed), Instituto de Salud Carlos III, 28031 Madrid, Spain; 3 Department of Biochemistry and Physiology, Faculty of Pharmacy and Food Sciences, University of Barcelona, 08028 Barcelona, Spain; alilloma55@gmail.com; 4 Department of Pharmacology, Toxicology and Therapeutic Chemistry, Institute of Biomedicine (IBUB), University of Barcelona, 08028 Barcelona, Spain; raullopezarnau@ub.edu (R.L.-A.); jcamarasa@ub.edu (J.C.); eescubedo@ub.edu (E.E.); 5 Chemistry School, Department of Biochemistry and Molecular Biomedicine, University of Barcelona, 08028 Barcelona, Spain

**Keywords:** G protein-coupled receptor GPCR, striatal neurons, heteromer, drug of abuse, neuroprotection

## Abstract

Methamphetamine is, worldwide, one of the most consumed drugs of abuse. One important side effect is neurodegeneration leading to a decrease in life expectancy. The aim of this paper was to check whether the drug affects one of the receptors involved in neurodegeneration/neuroprotection events, namely the adenosine A_2A_ receptor (A_2A_R). First, we noticed that methamphetamine does not affect A_2A_ functionality if the receptor is expressed in a heterologous system. However, A_2A_R becomes sensitive to the drug upon complexes formation with the cannabinoid CB_1_ receptor (CB_1_R) and the sigma 1 receptor (σ_1_R). Signaling via both adenosine A_2A_R and cannabinoid CB_1_R was affected by methamphetamine in cells co-expressing the two receptors. In striatal primary cultures, the A_2A_R–CB_1_R heteromer complex was detected and methamphetamine not only altered its expression but completely blocked the A_2A_R- and the CB_1_R-mediated activation of the mitogen activated protein kinase (MAPK) pathway. In conclusion, methamphetamine, with the participation of σ_1_R, alters the expression and function of two interacting receptors, A_2A_R, which is a therapeutic target for neuroprotection, and CB_1_R, which is the most abundant G protein-coupled receptor (GPCR) in the brain.

## 1. Introduction

Methamphetamine is one of the most consumed drugs of abuse in developed countries. It causes significant health and socio-economic problems that impact on sufferers, families, and the civil Society as a whole. Cocaine and methamphetamine share their ability to increase the brain levels of one of the main neurotransmitters, dopamine. Seemingly, the two drugs share some of the mechanisms that lead to addiction, but they also display differential trends.

Five dopamine receptors have been identified so far: D_1_, D_2_, D_3_, D_4_ and D_5_. They belong to the superfamily of G protein-coupled receptor (GPCRs) and, in mammals, they are expressed in neural cells but also in many other cell types. In the central nervous system, these receptors mediate the effects of dopamine in almost any higher function: from cognition to motor control. Plastic changes affecting the expression of dopamine receptors in the neurons of the reward circuits are concomitant with drug addiction and relapse. Dopamine action is counterbalanced by adenosine, a neuromodulator whose receptors, A_1_, A_2A_, A_2B_ and A_3_, in addition to being GPCRs, are widely distributed in the central nervous system (CNS). Among adenosine receptors, A_2A_ is arising as a target for neuroprotection. There are several studies, both in vitro and in animal models of neurodegenerative diseases, that show that antagonists of this receptor have neuroprotective potential. These results add to the epidemiological finding that natural antagonists of adenosine receptors, such as caffeine, theophylline or theobromine, reduce the risk of suffering from Parkinson’s or Alzheimer’s diseases [1,2,3,4,5,6,7,8,9].

In the basal ganglia, dopamine and adenosine receptors interact to form functional complexes. Interestingly, we have previously discovered a functional complex that mediates the motor effects of cannabinoids formed by A_2A_, D_2_ and a cannabinoid receptor, CB_1_ [10,11,12]. Endocannabinoids are neuromodulators acting on two GPCRs, cannabinoid CB_1_ and CB_2_ receptors (CB_1_R and CB_2_R), which are expressed in different systems of the mammalian body. Some effects of natural phytocannabinoids are mediated by these receptors, that are differentially expressed in the CNS: the CB_1_ is more abundant in neurons and the CB_2_ is more abundant in non-neuronal cells. Although trans(-)Δ^9^-tetrahydrocannabinol (Δ^9^-THC), the most well-known compound of *Cannabis sativa*, has psychotropic effects, its potential to induce addiction is controversial. Accordingly, the role of cannabinoid receptors in addiction is not well understood.

Relevant for this project was (i) the discovery that Δ^9^-THC blocks methamphetamine-induced neurotoxicity [13,14] and (ii) the recent discovery that an allosteric modulator of the CB_1_R, cannabidiol [15], suppresses the cognitive effect of Δ^9^-THC via the A_2A_/CB_1_ receptor heteromer [16].

It is known that, to exert some of its noxious effects, cocaine binds to the sigma 1 receptor (σ_1_R), which does not belong to the GPCR family and has no known endogenous agonist [17,18,19,20,21]. Intriguingly, methamphetamine alters the functionality of the σ_1_R, although the underlying mechanism remains obscure [22,23,24,25,26]. The aim of this paper was to investigate whether methamphetamine, in a σ_1_R-dependent or -independent fashion, was able to alter the interaction and functionality of the heteromer formed by A_2A_ and CB_1_ receptors (A_2A_–CB_1_Het) in both a heterologous expression system and primary cultures of striatal neurons.

## 2. Results

### 2.1. Methamphetamine Does Not Disrupt the Formation of A_2A_ and CB_1_ Receptor Complexes in a Heterologous Expression System

**Figure 1:**
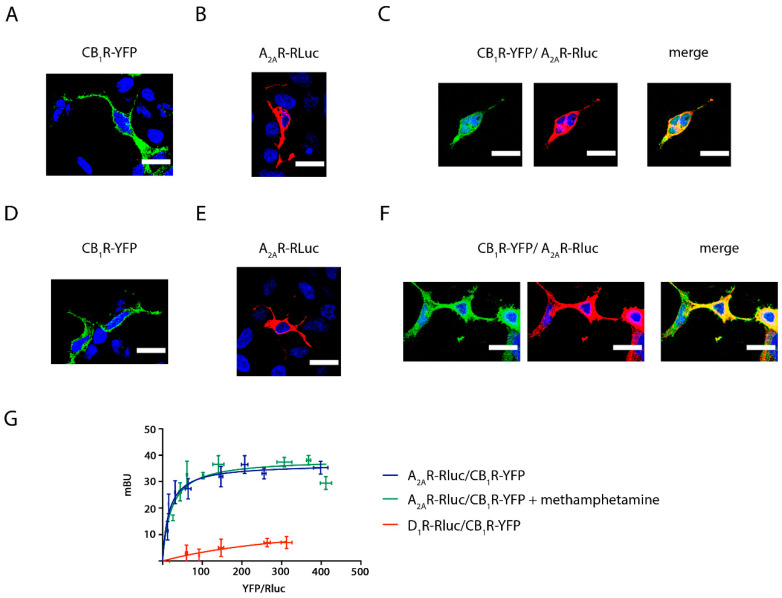
The effect of methamphetamine on A_2A_R–CB_1_R colocalization and receptor–receptor interaction. Confocal microscopy images were obtained in HEK-293T cells transfected with 0.5 μg cDNA coding for CB_1_R-YFP (**A**,**D**), with 0.5 μg cDNA for A_2A_R-Rluc (**B**,**E**) or with 0.5 μg cDNA for both CB_1_R-YFP and A_2A_R-Rluc (**C**,**F**). Cells were treated with 1 μM methamphetamine (**D**–**F**) or vehicle (**A**–**C**) for 1 h. Receptors fused to Rluc (red) were identified by immunocytochemistry and receptors fused to YFP (green) were identified by their own fluorescence. Colocalization is shown in yellow. Nuclei were stained with Hoechst (blue). Images are taken near the surface of the slide to observe a higher portion of the plasma membrane. Scale bars: 20 μm. (**G**): Bioluminescence Resonance Energy Transfer (BRET) assays were performed in HEK-293T cells transfected with a constant amount of the cDNA coding for A_2A_R fused to Rluc (1 μg) or the cDNA coding for D_1_R fused to Rluc (0.5 μg to 4.5 μg) (red line) and increasing amounts of the cDNA coding for CB_1_R fused to YFP (0.5 μg to 4.5 μg). Transfected cells were treated (green dots) or not (blue dots) with 1 μM methamphetamine. BRET is expressed as milli BRET units (mBU) and is given as the mean ± SEM of 8 different experiments grouped by amount of BRET acceptor.

The effect of methamphetamine on receptor expression was first assessed in transfected HEK-293T cells. Immunocytochemistry assays showed that the membrane expression level of CB_1_R was not altered when cells were pre-treated for 1 h with methamphetamine (1 μM) (Figure 1A,D). Similar results were observed in HEK-293T cells expressing A_2A_R (Figure 1B,E). When analyzing HEK-293T cells expressing equal amounts of CB_1_R and A_2A_R, the two receptors colocalized at the plasma membrane level (yellow in Figure 1C) and methamphetamine pretreatment did not induce any significant change in colocalization (Figure 1F).

A closer inspection using bioluminescence resonance energy transfer (BRET) showed a saturation curve, thus confirming the formation of complexes of A_2A_R-Rluc and CB_1_R-YFP (BRET_max_ = 37 ± 2; BRET_50_ = 17 ± 4), and a lack of effect of methamphetamine (BRET_max_ = 39 ± 3; BRET_50_ = 22 ± 9). A linear relationship was found for the negative control performed using plasmids encoding for receptors that do not heteromerize, D_1_R-Rluc and CB_1_R-YFP (Figure 1G).

### 2.2. Methamphetamine Blocks CB_1_R Function in HEK-293T Cells Expressing the A_2A_–CB_1_Het

HEK-293T cells transfected with the cDNA for A_2A_ and CB_1_ receptors, i.e., expressing heteromers formed by the two receptors (A_2A_–CB_1_Hets), were pre-treated with selective antagonists (SCH 58261 for A_2A_R or SR 141716A for CB_1_R) prior to treatment with selective agonists (CGS 21680 for A_2A_R and/or arachidonyl-2′-chloroethylamide -ACEA- for CB_1_R) and the following signaling outputs were analyzed: cytosolic cAMP and Ca^2+^ level determination, ERK1/2 and Akt phosphorylation, β-arrestin 2 recruitment and dynamic mass redistribution (DMR).

**Figure 2:**
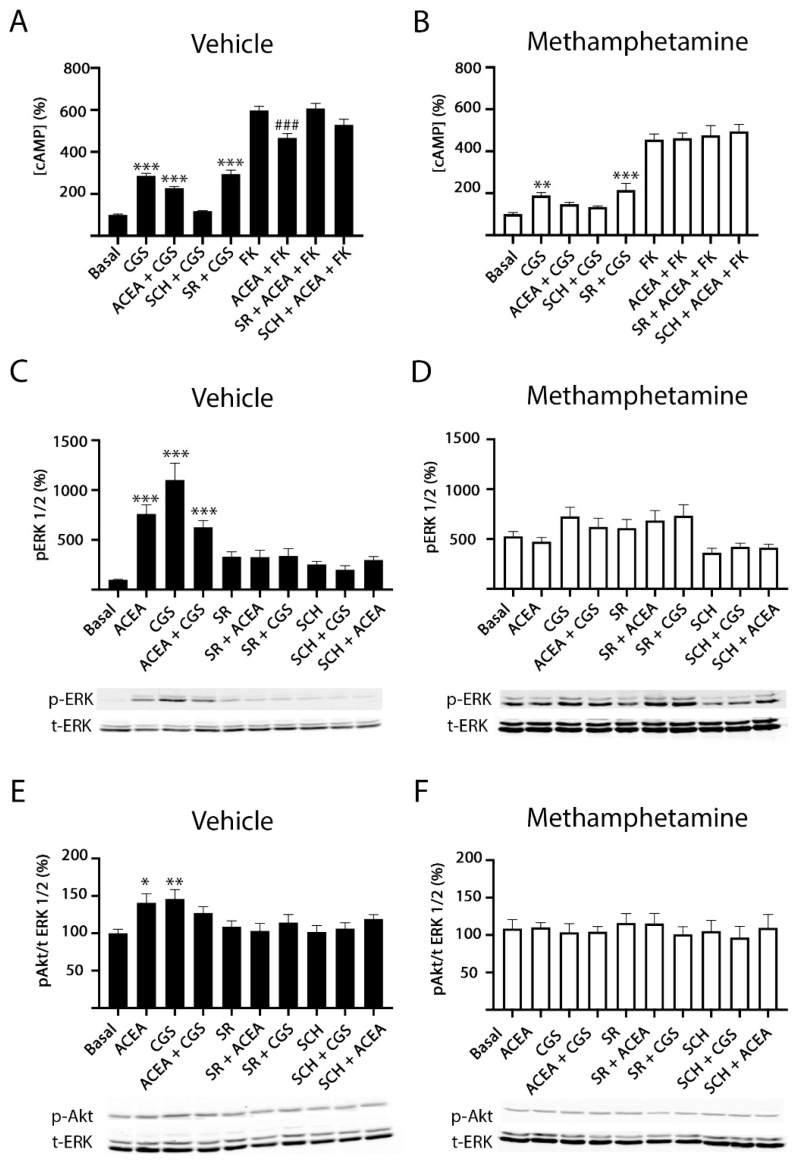
Methamphetamine effect on A_2A_–CB_1_Het functionality: cAMP and ERK/Akt phosphorylation. HEK-293T cells co-transfected with cDNAs for A_2A_R (0.5 µg) and for CB_1_R (0.6 µg) were treated (30 min) with vehicle (**A**,**C**,**E**) or 1 µM methamphetamine (**B**,**D**,**F**) and subsequently treated with 500 nM of the A_2A_R antagonist SCH 58261 (SCH) or with 500 nM of the CB_1_R antagonist SR 141716A (SR). Receptors were activated using 100 nM of the A_2A_R agonist CGS 21680 (CGS), with 200 nM of the CB_1_R agonist, ACEA or both, before adding 0.5 µM forskolin (FK) or vehicle. The cytosolic cAMP levels (**A**,**B**) and the extracellular signal-regulated (ERK) 1/2 (**C**,**D**) or Akt (**E**,**F**) phosphorylation signals were determined. Results are expressed as percentage over basal and are the mean ± SEM of six experiments performed in triplicates. A one-way ANOVA followed by Bonferroni multiple comparison post hoc test showed a significant effect (* *p* < 0.05, ** *p* < 0.01, *** *p* < 0.001 vs. basal condition; ### *p* < 0.001 vs. FK condition). In (**C**–**F**) a representative Western blot is shown (bottom). *p*-ERK indicates phosphorylated ERKs, t-ERK indicates total ERKs, *p*-AKT indicates phosphorylated AKTs.

Due to A_2A_R coupling to the Gs protein, which activates adenylyl cyclase, and CB_1_R coupling to the Gi protein, which inhibits adenylate cyclase, we analyzed the effect of agonists, alone or in combination, in naïve cells and in cells pretreated with forskolin (0.5 µM, 15 min, FK), the activator of the adenylyl cyclase. The A_2A_R agonist, CGS 21680, induced a significant increase in cAMP (around 3-fold compared to basal condition), while treatment with the CB_1_R agonist, ACEA, induced a significant decrease over forskolin cAMP level increases (Figure 2A). These results indicate that both A_2A_R and CB_1_R can be activated when forming A_2A_R–CB_1_R complexes and are able to couple to their respective cognate G proteins. An often-found feature of heteromers is cross-antagonism, i.e., the antagonist of one receptor also blocks the signal mediated by the partner receptor within the heteromer. When cotransfected cells were pre-treated with the selective antagonists before agonist stimulation, it was observed that the A_2A_R antagonist counteracted A_2A_R signaling, while it had a slight effect over CB_1_R activation. A qualitatively similar result was obtained using the CB_1_R antagonist, i.e., blockade of activation of the cognate receptor, CB_1_R, but not of A_2A_R activation (Figure 2A). When a similar experiment was undertaken in cells treated for 1 h with methamphetamine before stimulation with selective agonists, CB_1_R signaling was negligible (Figure 2B). After methamphetamine action, cross-antagonism was still undetectable. It should be noted that ACEA does not affect basal cAMP production and that, at the concentration used, CGS does not affect forskolin-induced cAMP production.

In cells expressing A_2A_–CB_1_Hets, both agonists, CGS 21680 and ACEA, increased ERK1/2 and Akt phosphorylation. Moreover, when the same cells were simultaneously coactivated with both agonists, the effect was lower than the sum of individual effects at the same ligand concentration (Figure 2C,E). Interestingly, the A_2A_R antagonist not only blocked the CGS 21680- but also the ACEA-induced effect. Such cross-antagonism was also detected when cells were treated with the selective CB_1_R antagonist. Under the same assay conditions, pretreatment with methamphetamine completely blocked CB_1_R function and decreased A_2A_R-mediated function (Figure 2D,F).

**Figure 3:**
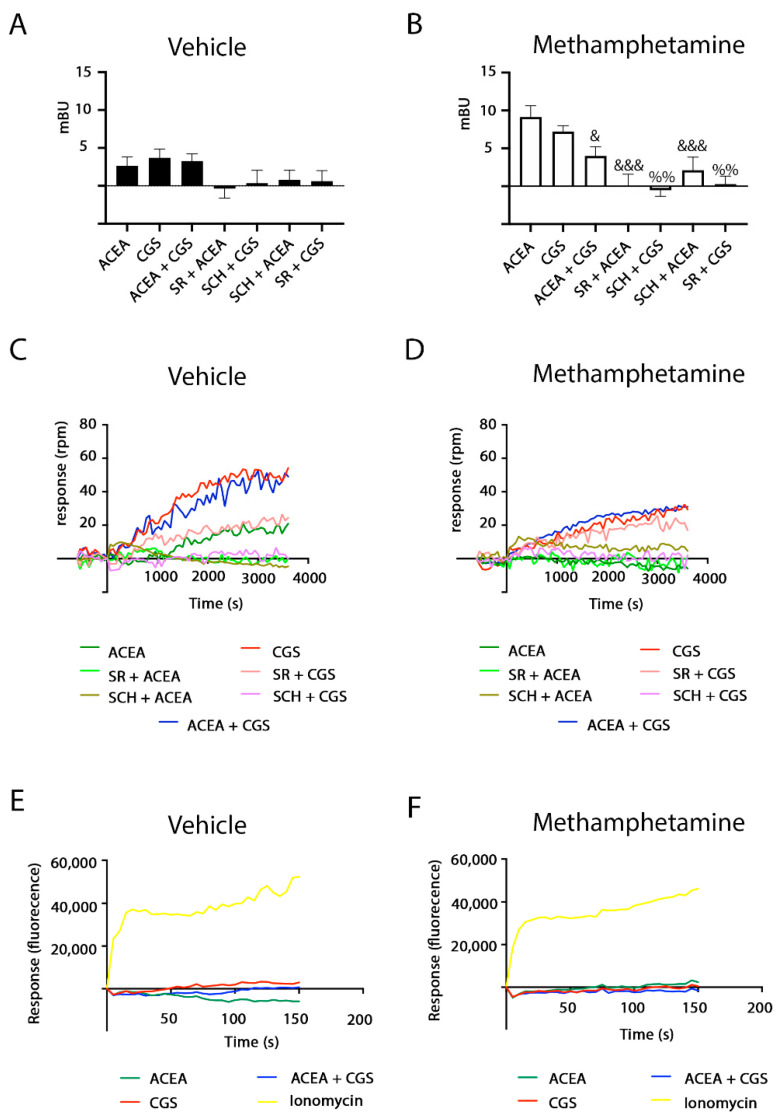
Methamphetamine effect on A_2A_–CB_1_Het functionality: ß-arrestin-2 recruitment, dynamic mass redistribution (DMR) and Ca^2+^ mobilization. HEK-293T cells transfected with cDNAs corresponding to A_2A_R-YFP (0.4 µg), CB_1_R (0.3 µg) and ß-arrestin-2-Rluc (0.5 µg) (**A**,**B**) or with A_2A_R (0.5 µg), CB_1_R (0.6 µg) (**C**,**D**) and 6GCaMP calcium sensor (1.5 µg) (**E**,**F**) were treated (30 min) with vehicle (**A**,**C**,**E**) or with 1 µM methamphetamine (**B**,**D**,**F**) and subsequently treated with 500 nM of the A_2A_R antagonist SCH 58261 (SCH) or with 500 nM of the CB_1_R antagonist SR 141716A (SR). Receptors were activated using 100 nM of the A_2A_R agonist CGS 21680 (CGS), with 200 nM of the CB_1_R agonist ACEA or both. In (**E**,**F**), a portion of the cells were stimulated with 1 µM ionomycin as a positive control. The BRET data resulting from ß-arrestin-2 recruitment (**A**,**B**), time-dependent DMR representative traces (**C**,**D**) and time-dependent representative traces of cytoplasmic Ca^2+^ levels (**E**,**F**) are shown. In (**A**,**B**), BRET is expressed as milli BRET units (mBU) and is given as the mean ± SEM of six different experiments grouped by amount of BRET acceptor. A one-way ANOVA followed by Bonferroni multiple comparison post hoc test showed a significant effect over 100% (& *p* < 0.05, &&& *p* < 0.001 versus ACEA condition; %% *p* < 0.01 versus CGS condition).

After receptor activation, most GPCRs recruit β-arrestin before internalization. Interestingly, when analyzing β-arrestin recruitment in HEK-293T cells coexpressing A_2A_R and CB_1_R, it was observed that recruitment was very small (Figure 3A). However, β-arrestin recruitment was potentiated in cells pretreated with methamphetamine (Figure 3B).

Dynamic mass redistribution (DMR) is a technique that allows detection of G protein-mediated signaling in living cells by measuring cytoskeleton movements derived from receptor activation [27,28,29,30]. Selective agonists led to a significant signal although simultaneous treatment with agonists did not lead to summation of effects. The use of the output signal in the presence of antagonists underscored cross-antagonism (Figure 3C). Analysis by DMR of A_2A_R activation showed that it was partially inhibited by methamphetamine pre-treatment, while CB_1_R activation was completely blocked by the drug of abuse (Figure 3D).

In agreement with A_2A_R coupling to Gs and CB_1_R coupling to Gi protein, neither activation of A_2A_R, nor of CB_1_R led to significant alterations in cytoplasmic Ca^2+^ levels (Figure 3E). Although some drugs are able to produce a shift in the G protein-coupled to a given GPCR, methamphetamine did not promote Gq coupling to A_2A_R or to CB_1_R receptors (Figure 3F).

### 2.3. Methamphetamine Action in Cells Expressing A_2A_ or CB_1_ Receptors

**Figure 4:**
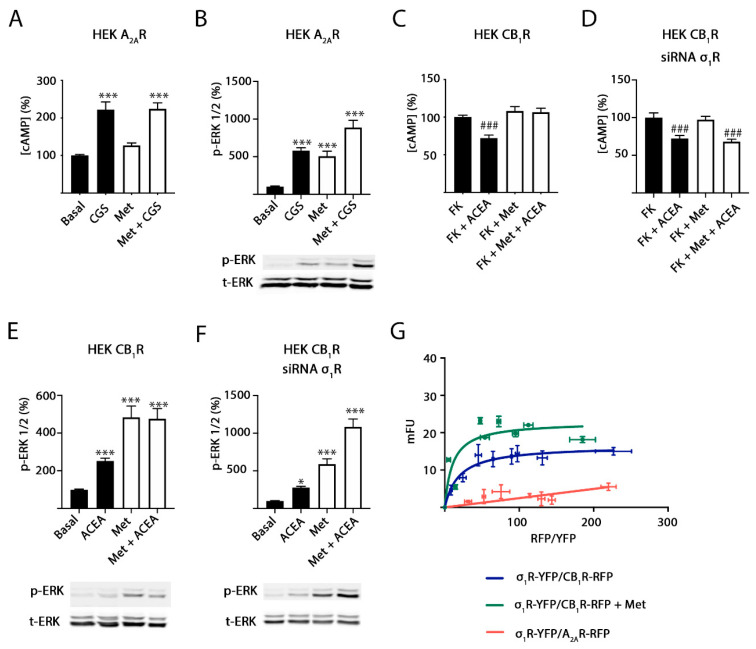
σ_1_R involvement in methamphetamine effects on A_2A_ and CB_1_ receptors. HEK-293T cells transfected with cDNAs coding for A_2A_R (0.5 µg) (**A**,**B**) or for CB_1_R (0.6 µg) (**C**–**F**) in the absence (**A**–**C**,**E**) or presence (**D**,**F**) of siRNA for σ_1_R (3 μg) to silence σ_1_R were stimulated with 1 µM methamphetamine (Met, white bars) or vehicle (black bars) for 30 min. Cells were treated with 100 nM of the A_2A_R agonist CGS 21680 (CGS) (**A**,**B**) or with 200 nM of the CB_1_R agonist, ACEA (**C**–**F**). 0.5 μM of forskolin (FK) was used to determine Gi coupling to the CB_1_R. Then, cAMP levels (**A**,**C**,**D**) and ERK1/2 phosphorylation (**B**,**E**,**F**) were determined and expressed as percentage over basal or, in the case of cAMP in CB_2_R-expressing cells, over forskolin treatment. Values are the mean ± SEM (*n* = 5, in triplicates). One-way ANOVA followed by Bonferroni multiple comparison post hoc test showed a significant effect (* *p* < 0.05, *** *p* < 0.001 vs. basal condition, ### *p* < 0.001 versus FK condition). In (**B**,**E**,**F**), a representative Western blot is shown (bottom). In (**G**), FRET experiments were performed in HEK-293T cells transfected with 0.25 μg of cDNA for σ_1_R-YFP and increasing amounts of cDNA for CB_1_R-RFP (0.2 μg to 2 μg) or for A_2A_R-RFP (0.1 μg to 1.5 μg). When indicated cells were pretreated with 1 µM methamphetamine. Results were grouped by amount of FRET acceptor. *p*-ERK indicates phosphorylated ERKs, t-ERK indicates total ERKs.

It should be noted that the σ_1_R, which mediates the action of some drugs of abuse, is endogenously expressed in HEK-293T cells [20,21,31,32]. Hence, we next addressed the mechanisms by which methamphetamine alters signaling in cells expressing A_2A_ or CB_1_ receptors. We first analyzed data from HEK-293T cells expressing only the A_2A_R. In cAMP level determination assays, the CGS 21680-induced increase in cAMP levels was not affected by pretreatment with methamphetamine (Figure 4A). This result was different from that observed in Figure 2A,B, where in HEK-293T cells expressing A_2A_–CB_1_Hets, methamphetamine pretreatment partially blocked A_2A_R signaling. Data from MAPK pathway activation shows that methamphetamine per se is able to activate this signaling pathway; combined treatment of the drug of abuse and CGS 21680 resulted in an additive-like response, i.e., the drug did not disrupted the link of A_2A_R activation to the MAPK pathway (Figure 4B). Hence, it appears that the action of methamphetamine on the A_2A_R depends on CB_1_R expression and, likely, on A_2A_–CB_1_Het expression.

In contrast, the effect of methamphetamine on CB_1_R-mediated signaling is not dependent on A_2A_R expression. In fact, ACEA effects on forskolin-induced increases of cAMP levels (Figure 4C) and on MAPK pathway activation (Figure 4E) were blocked by methamphetamine. To further explain the mechanism by which methamphetamine may block CB_1_R action, we focused on the σ_1_ receptor (σ_1_R), which is a transmembrane receptor that does not belong to the GPCR family. It is a target of cocaine and its functionality in rodents is altered upon methamphetamine treatment [22,26,33]. Interestingly, the methamphetamine blockade of CB_1_R signaling was mediated by the σ_1_R as it disappeared when a siRNA designed for σ_1_R silencing was used (Figure 4D,F). In terms of ERK phosphorylation, the methamphetamine action was not further increased by the activation of the CB_1_R (Figure 4E). However, a summation effect was obtained in cells in which the σ_1_R was silenced (Figure 4F). Altogether, the results suggest that the σ_1_R mediates the inhibition of the CB_1_R to the MAPK and cAMP pathways exerted by methamphetamine and that σ_1_R and CB_1_R could physically interact. We performed fluorescence resonance energy transfer (FRET) assays using cells expressing a constant amount of cDNA for σ_1_R-YFP and increasing amounts of cDNA for CB_1_R-RFP. The interaction is possible as deduced from the saturation curve (Figure 4G). FRET parameters were: FRET_max_ = 17 ± 1 and FRET_50_ = 19 ± 5. The interaction was maintained in cells pretreated with methamphetamine although with a significant alteration in FRET parameters (FRET_max_ = 23 ± 3, FRET_50_ = 10 ± 5). On the one hand, these results indicate that the drug produces an increase in CB_1_R-σ_1_R complexes or a structural reorganization leading to an approximation of the two fluorescent proteins. On the other hand, in agreement with the lack of effect of methamphetamine on the A_2A_R when the CB_1_R is not present, FRET assays showed a linear nonspecific signal, i.e., a lack of interaction between σ_1_R-YFP and A_2A_R-RFP (Figure 4G, red line).

### 2.4. σ_1_ Receptor Involvement in Methamphetamine Action in Cells Expressing A_2A_–CB_1_Hets

**Figure 5:**
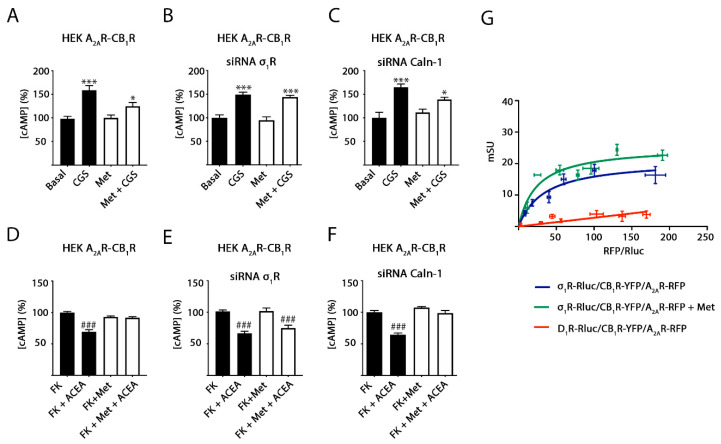
σ_1_R involvement in methamphetamine effects on A_2A_–CB_1_Hets. HEK-293T cells were co-transfected with cDNAs coding for A_2A_R (0.5 μg) and for CB_1_R (0.6 μg) (**A**–**F**). When indicated, σ_1_R (**B**,**E**) or calneuron-1 (Caln-1) (**C**,**F**) were silenced using 3 μg specific siRNA. Cells were pretreated with 1 μM methamphetamine (Met, white bars) or vehicle (black bars) for 30 min. GPCRs were activated either using 100 nM of the A_2A_R agonist, CGS 21680 in naïve cells (CGS, (**A**–**C**)) or 200 nM of the CB_1_R agonist, ACEA, in cells treated with 0.5 μM of forskolin (FK) (**D**–**F**). Then, cAMP levels (**A**–**F**) were determined and expressed as a percentage over basal (A_2A_R) or forskolin (CB_1_R). Values are the mean ± SEM of five different experiments performed in triplicates. One-way ANOVA followed by Bonferroni multiple comparison post hoc test showed significant effects (* *p* < 0.05, *** *p* < 0.001 vs. basal condition or ### *p* < 0.001 vs. FK condition). In (**G**), SRET experiments were performed in HEK-293T cells transfected with constant amounts of cDNAs for σ_1_R-Rluc (0.5 μg) or D_1_R-Rluc (0.33 μg) and for CB_1_R-YFP (1 μg) and increasing amounts of cDNA for A_2A_R-RFP (0.25 μg to 2.5 μg). Results were grouped by amount of SRET acceptor.

Experiments similar to those described in the previous section were performed in cotransfected cells, that is, in cells expressing A_2A_–CB_1_Hets. Figure 5A shows that the activation of the A_2A_R with CGS 21680 was significantly altered by methamphetamine pretreatment. Therefore, alteration of A_2A_R-mediated signaling is dependent upon CB_1_R expression and A_2A_–CB_1_ receptor heteromerization (Figure 5). Furthermore, the σ_1_R was required as the effect of the drug on A_2A_R-mediated signaling disappeared in cells in which the σ_1_R was silenced (Figure 5B) but not in cells treated with a siRNA designed for calneuron-1 silencing (Figure 5C). These results suggest that a functional complex formed by the three receptors is expressed in these cells. In contrast, the results obtained using the agonist of the CB_1_R were similar to those found in cells only expressing the cannabinoid receptor (Figure 5D–F). To test the hypothesis of trimer formation, we took advantage of a technique that allows to detect interactions of three different proteins, namely sequential resonance energy transfer (SRET). These experiments were performed in HEK-293T cells expressing σ_1_R-Rluc, CB_1_R-YFP and transfected with increasing amounts of cDNA for A_2A_R-RFP. Saturation of the SRET curve (Figure 5G) indicates that the three proteins interact (the dopamine D_1_ receptor was used as a negative control). The SRET signal did not significantly change upon treatment with methamphetamine (SRET_max_ = 21 ± 2 and SRET_50_ = 34 ± 12 in the absence of the drug and SRETmax = 25 ± 3 and SRET_50_ = 24 ±10 in the presence of methamphetamine). As the A_2A_R is unable to interact with σ_1_R (Figure 4G), it is hypothesized that the trimer is formed by the CB_1_R ability to bind to both A_2A_R and σ_1_R.

### 2.5. CB_1_R-σ_1_R Expression Is Altered When Striatal Primary Cultures of Neurons Are Treated with Methamphetamine

**Figure 6:**
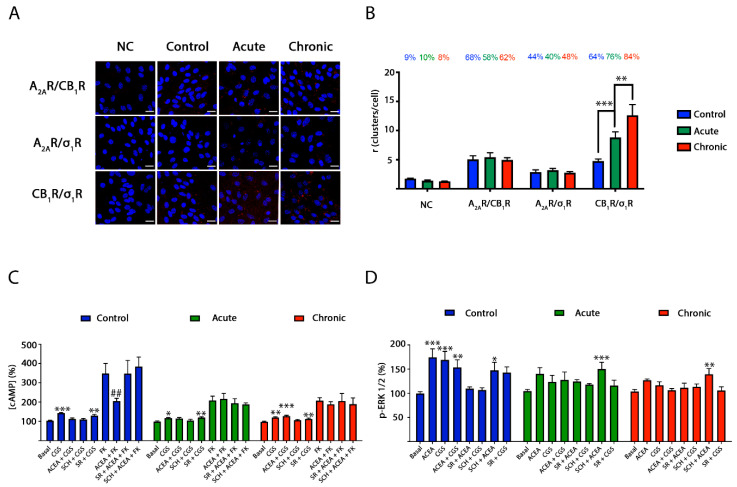
Methamphetamine effect on A_2A_–CB_1_Het expression and signaling in striatal neurons. (**A**,**B**) Proximity ligation assays (PLA) were performed in striatal neurons treated with 1 µM methamphetamine for two hours (acute, green bars), for one week (chronic, red bars) or with vehicle (control, blue bars) using primary antibodies specific for the A_2A_, σ_1_ and CB_1_ receptors. As a negative control (NC), neurons were only treated with the primary antibody against CB_1_R. See Methods for details on the steps in the PLA procedure. Confocal microscopy images (stacks of 4 consecutives planes) show heteroreceptor complexes as red clusters over Hoechst-stained nuclei (blue). Scale bar 20 µm (**A**). The bar graph (**B**) shows the number of red dots/neuron and the number above the bars indicates the percentage of neurons presenting red dots. Values are the mean ± SEM (*n* = 8–10). A two-way ANOVA followed by Bonferroni’s multiple comparison post hoc test were used for statistical analysis (** *p* < 0.01, *** *p* < 0.001). cAMP levels (**C**) and ERK 1/2 phosphorylation (**D**) were determined in striatal neurons treated with methamphetamine 1 µM for two hours (acute, green bars) or for one week (chronic, blue bars) or vehicle (control, blue bars). When indicated cells were incubated with 500 nM of the A_2A_R antagonist SCH 58261 (SCH) or with 500 nM of the CB_1_R antagonist SR 141716A (SR). Receptors activated using 100 nM of the A_2A_R agonist CGS 21680 (CGS) or with 200 nM of the CB_1_R agonist ACEA. Forskolin (FK) was used to determine Gi coupling to the CB_1_ receptor (**C**). The values are the mean ± SEM of six experiments performed in triplicates. A two-way ANOVA followed by Bonferroni’s multiple comparison post hoc test were used for statistical analysis (* *p* < 0.05, ** *p* < 0.01, *** *p* < 0.001 versus basal condition; ## *p* < 0.01 versus FK condition).

The in situ proximity ligation assay (PLA) is instrumental in detecting in natural sources complexes formed by two membrane proteins [34,35]. PLA was determined in primary cultures of striatal neurons. Heteromers are detected as red dots surrounding Hoechst-stained nuclei. Sixty-eight percent of neurons expressed red dots when analyzing CB_1_R–A_2A_R complexes by using anti-A_2A_R and anti-CB_1_R antibodies, with around five red dots per cell expressing dots (Figure 6A,B). Moreover, when analyzing CB_1_R–σ_1_R complexes, similar values were observed: 64% of neurons showed red clusters with around five red dots per cell expressing dots. When A_2A_R–σ_1_R complexes were analyzed, 44% of neurons showed red clusters with around 3 red dots per cell expressing dots. Due to the fact that A_2A_R does not interact with σ_1_R (Figure 4G), it seems likely that a significant amount of neurons are expressing A_2A_–CB_1_–σ_1_R heterotrimers. Unfortunately, PLA is unable to detect complexes formed by three proteins.

PLA experiments were also performed in striatal neurons treated with 1 µM methamphetamine for two hours (acute) or for one week (chronic). On the one hand, the drug in either acute or chronic treatment did not affect the expression of A_2A_R–CB_1_R and of A_2A_R–σ_1_R complexes. On the other hand, the expression of CB_1_R–σ_1_R complexes was significantly increased upon acute treatment (76% of neurons expressing red dots and around 10 red dots per cell expressing dots) and even more after the chronic regime (84% of neurons expressing red dots and around 13 red dots per cell expressing dots) (Figure 6A,B).

### 2.6. Blockade by Methamphetamine of CB_1_R–A_2A_R Complex Signaling in Striatal Neurons

First, in signaling assays performed in striatal neurons, the A_2A_–CB_1_Het print, namely cross-antagonism, was detected (Figure 6). In addition, the non-additive effect observed in the heterologous expression system was detected (Figure 6). After demonstrating A_2A_–CB_1_Het expression by PLA (see previous section) and by detection of the heteromer print, further assays were performed to evaluate how methamphetamine may affect signaling in these cells. Primary cultures of striatal neurons were treated for two hours (acute) or for one week (chronic) with methamphetamine prior to assessing the effect of A_2A_R or CB_1_R agonists on cAMP levels and MAPK pathway activation. Interestingly, it was first observed that ACEA-induced decreases of cAMP levels were partially inhibited by acute and chronic methamphetamine treatments (Figure 6C), while ERK1/2 phosphorylation was completely blocked (Figure 6D). Similarly, CGS 21680-induced increases in cAMP levels were partially blocked after acute and chronic methamphetamine treatments, while ERK1/2 phosphorylation was completely blunted (Figure 6C,D). These results show that the drug, likely via interaction with σ_1_R in a heteromer context, partially reduces G-protein-mediated signaling while completely abolishing MAPK pathway activation. Interestingly, the effect on A_2A_R signaling to the MAPK pathway did not occur in the presence of a CB_1_R antagonist.

## 3. Discussion

Cannabinoids and adenosine are two important neuromodulators in the central nervous system (CNS). Two of their receptors have attracted interest due to their potential as therapeutic targets. On the one hand, the CB_1_R is, reportedly, the most abundant GPCR in the CNS and is mainly expressed in neurons [36,37,38]. Its potential is limited as some of their agonists, such as the natural *Cannabis sativa*-derived compound Δ^9^-THC, lead to psychotropic effects, while its antagonists may have serious side effects [39]. On the other hand, the A_2A_R receptor is heavily expressed in the brain striatum where it regulates dopamine neurotransmission. In addition, (i) it is expressed in other brain areas, in both neurons and glia [40], and (ii) there is cumulative evidence of its participation in neurodegenerative/neuroprotection events. Importantly, a first in class A_2A_R antagonist, istradefylline, has been approved for the therapy of Parkinson’s disease (Nouriast^TM^ in Japan and Nourianz^TM^ in the USA) [41,42,43,44,45,46]. This approval puts the A_2A_R on the front line to combat neurodegeneration and/or to afford neuroprotection.

The mechanisms of neurodegeneration due to methamphetamine consumption are not well understood yet. It should be noted that the σ_1_R is a common factor in cocaine and methamphetamine addiction [26]. It is well established that cocaine binds to this atypical transmembrane receptor, which is structurally different to GPCRs, occurs as a homotrimer and has no known endogenous agonist [47,48,49]. In animals treated with methamphetamine, the alterations involve σ_1_R-mediated events. About 15 years ago it was reported that locomotor effects of the drug could be reverted if animals were administered antisense probes that reduced the expression of the σ_1_R receptor in the brain; similar results were obtained using the commercially available antagonists [22]. Our first hypothesis, namely that the σ_1_R could interact with the A_2A_R and mediate effects of the drug on the receptor was not proven. Not only did the two receptors not interact, but methamphetamine did not directly affect A_2A_R functionality. However, CB_1_R is required for methamphetamine altering A_2A_R signaling.

Previous experience has shown us that the σ_1_R may interact with GPCRs to form complexes that mediate some of the relevant central effects exerted by cocaine. For instance, an interaction between σ_1_, orexin and corticotropin-releasing factor receptors are the target of cocaine in the ventral tegmental area [50]. Remarkably, the anorexic effect of cocaine is, at least in part, due to the formation of a complex between the σ_1_R and the ghrelin receptor that allows the blockade of ghrelin action [31]. Our next hypothesis was then to consider interactions of the σ_1_R with GPCR heteromers, whose functionality is different from that exerted by individual receptors [51,52,53]. In fact, biased signaling may be achieved by GPCR heteromers and/or by complexes formed by GPCRs and other proteins [54]. We here demonstrate that σ_1_R may interact with the CB_1_R so that σ_1_R, CB_1_R and A_2A_R may form trimers.

Although the A_2A_R may interact with several other GPCRs (see www.gpcr-hetnet.com/, accessed on 7 March 2021), we were interested in interactions with the receptors of cannabinoids and, in particular, with the most abundant GPCR among CNS neurons, the CB_1_R. Actually, the interaction of these two GPCRs in an heterologous expression system and, among others, in the striatum and in CA1 neurons in the hippocampus, has been reported [10,16,55,56,57,58]. Then, we aimed at finding whether the σ_1_R could interact with the CB_1_R and, upon confirming this possibility, whether methamphetamine acting on σ_1_R could alter A_2A_R-mediated signaling via the A_2A_–CB_1_Het. The results not only confirmed the hypothesis but showed a strong effect upon the A_2A_R in co-transfected HEK-293 cells and in primary cultures of striatal neurons. Alterations by the drug in cAMP-dependent signaling are important but less than those in the MAPK pathway. In fact, the A_2A_–CB_1_Het is coupled to both Gs and Gi and both are altered, thus leading to a counterbalancing effect. In contrast, the effect of the drug on the MAPK pathway was in the same direction for both receptors, i.e., blocking the activation of the pathway. In addition, the results indicate that methamphetamine acts on a functional unit formed by the three receptors but with the A_2A_R being unable to bind to σ_1_R and with the CB_1_R able to bind both σ_1_ and A_2A_ receptors.

In summary, methamphetamine alters A_2A_R-mediated signaling in striatal neurons but only if the CB_1_R is present and the A_2A_–CB_1_Het is expressed. Failure to properly activate the MAPK signaling pathway in neurons unrolls the neurodegenerative mechanism due to the lack of those transcription factors that favor the expression of neuroprotective molecules (see [59] for review).

## 4. Materials and Methods

### 4.1. Reagents

Forskolin and receptor ligands CGS 21680 hydrochloride, ACEA, SCH 58261 and SR 141716A were purchased from Tocris Bioscience (Bristol, UK). Ionomycin and methamphetamine hydrochloride were purchased from MERK (St. Louis, MO, USA). siRNAs were purchased from ThermoFisher Scientific (Waltham, MA, USA).

### 4.2. Expression Vectors

cDNAs for the human version of cannabinoid CB_1_, adenosine A_2A_, dopamine D_1_ and σ_1_ receptors without the stop codon were obtained by PCR and subcloned to a Rluc-containing vector (pRluc-N1; PerkinElmer, Wellesley, MA, USA), to an enhanced yellow fluorescent protein-containing vector (pEYEP-N1; Clontech, Heidelberg, Germany) or to a cherry (RFP)-containing vector (pcDNA 3.1 Cherry) using sense and antisense primers harboring unique restriction sites for HindIII and BamHI to generate A_2A_R-Rluc, D_1_R-Rluc, CB_1_R-YFP, D_1_R-YFP, A_2A_R-RFP and CB_1_R-RFP or for BamHI and KpnI to generate σ_1_R-Rluc and σ_1_R-YFP fusion proteins.

### 4.3. Cell Culture and Transfection

HEK-293T cells were grown in Dulbecco’s modified Eagle’s medium (DMEM) supplemented with 2 mM L-glutamine, 100 U/mL penicillin/streptomycin, MEM Non-Essential Amino Acids Solution (1/100) and 5% (*v/v*) heat inactivated fetal bovine serum (FBS) (all from Gibco, Paisley, Scotland, UK). Cells were maintained in a humid atmosphere of 5% CO_2_ at 37 °C and were passaged when they were 80–90% confluent (number of passages < 18). Cells were transiently transfected with the corresponding cDNAs using the Polyethylenimine (PEI) (MERK, St. Louis, MO, USA) method as previously described [28,60] or with siRNA using the lipofectamine 2000 (ThermoFisher Scientific, Waltham, MA, USA) method following the instructions of the supplier cells were incubated with transfection reagents in serum-free medium; 4 h later the medium was replaced by complete medium. Experiments were carried out 48 h later.

To prepare mouse neuronal primary cultures, striatum from embryos (E19) was removed and neurons were isolated as described by Hradsky et al., 2013 [61]. In brief, striatum was digested with trypsin for 15 min at 37 °C. Trypsinization was stopped by several washes with HBSS (137 mM NaCl, 5 mM KCl, 0.34 mM Na_2_HPO_4_, 0.44 mM KH_2_PO_4_, 1.26 mM CaCl_2_, 0.4 mM MgSO_4_, 10 mM HEPES pH 7.4). Cells were brought to a homogeneous suspension by passage through 0.9 mm and 0.5 mm needles and through a 100 μm-pore mesh. Finally, cells were grown in neurobasal medium supplemented with 2 mM L-glutamine, 100 U/mL penicillin/streptomycin, MEM Non-Essential Amino Acids Solution (1/100) (all from Invitrogen, Paisley, Scotland, UK) and 2% (*v/v*) B27 supplement (Gibco, Paisley, Scotland, UK) for 12 days. For cAMP assays, cells were grown on 6-well plates at a density of 500,000 cells/well, for ERK 1/2 phosphorylation assays, cells were plated in 96-well plates at a density of 50,000 cells/well and for the proximity ligation assay, neurons were plated in coverslips in 12-well plates at a density of 100,000 cell/well. Cell counting was assessed using trypan blue and a countless II FL automated cell counter (ThermoFisher Scientific, Waltham, MA, USA).

The animal handling and protocols were conducted in accordance with the European Council Directive 2010/63/UE and the current Spanish legislation (RD53/2013). The ethics committee of the University of Barcelona were in charge of law implementation.

### 4.4. Immunocytochemistry

HEK-293T cells expressing A_2A_R-Rluc and/or CB_1_R-YFP seeded in coverslips were treated for one hour with vehicle or 1 µM methamphetamine, fixed in 4% paraformaldehyde for 15 min and washed twice with phosphate-buffered saline (PBS) containing 20 mM glycine to quench the aldehyde groups before permeabilization with PBS-glycine containing 0.2% Triton X-100 (5 min). Cells were treated for 1 h with PBS containing 1% bovine serum albumin (BSA), labelled with a primary mouse monoclonal anti-Rluc (1/100; EMD Millipore, Darmstadt, Germany, Ref. MAB4400) antibody for 1 h, and subsequently treated for 1 h with an anti-mouse Cy3-conjugated IgG (red) (1/200; Jackson Immuno Research, West Grove, PA, USA, Ref. 715-166-150) antibody. Nuclei were stained with Hoechst (1/100; MERK, St. Louis, MO, USA, Ref. B1155). Samples were washed several times and mounted with 30% Mowiol (Calbiochem, San Diego, CA, USA). CB_1_R-YFP expression was detected by YFP own fluorescence (green). Images were obtained in a Leica SP2 confocal microscope (Leica Microsystems, Mannheim, Germany) equipped with an apochromatic 63X oil-immersion objective (N.A. 1.4), and 405 nm, 488 nm and 561 nm laser lines.

### 4.5. Resonance Energy Transfer Assays

For bioluminescence resonance energy transfer (BRET) assays, HEK-293T cells were transiently co-transfected with constant amount of cDNAs encoding for receptor-Rluc fusion proteins and increasing amounts of cDNAs corresponding to receptor-YFP fusion proteins. Forty-eight hours post-transection, cells were detached using 0.1% glucose HBSS buffer and the cell suspension was adjusted to 20 µg/µL of protein using a Bradford assay kit (Bio-Rad, Munich, Germany) and BSA for standardization. Cells were treated with 1 µM methamphetamine or vehicle 1 h prior to each quantification. To quantify protein-YFP expression, cells were distributed in 96-well black plates with a clear bottom (Porvair, Wrexham, Wales, UK) and fluorescence was read in a Mithras LB 940 (Berthold Technologies, Bad Wildbad, Germany) equipped with a high-energy xenon flash lamp, using a 10-nm bandwidth excitation filter at 485 nm and 530 nm emission filter readings. For BRET measurements, cells were distributed in 96-well white plates with a clear bottom (Porvair) and readings were collected 1 min after the addition of 5 µM coelenterazine H (PJK GMBH, Kleinblittersdorf, Germany) using a Mithras LB 940, which allows the integration of the signals detected in the short-wavelength filter at 485 nm and the long-wavelength filter at 530 nm. To quantify protein-Rluc expression, luminescence readings were performed 10 min after 5 µM coelenterazine H addition using a Mithras LB 940 reader.

For fluorescence resonance energy transfer (FRET) assays, HEK-293T cells were transiently co-transfected with a constant amount of cDNAs encoding for receptor-YFP and increasing amounts of cDNAs corresponding to receptor-Cherry (RFP). Forty-eight hours after transfection, cells were detached using HBSS buffer with 0.1% glucose and cell suspension was adjusted to 20 µg/µL of protein using a Bradford assay kit (Bio-Rad, Munich, Germany) and bovine serum albumin (BSA) for standardization. Cells were treated with 1 µM methamphetamine or vehicle 1 h prior to each quantification. Cells were distributed in 96-well black plates with a clear bottom (Porvair). To quantify protein-YFP expression and protein-cherry expression, fluorescence was red in a FluoStar Optima Fluorimeter (BMG Labtechnologies, Offenburg, Germany) equipped with a high-energy xenon flash lamp, using a 10-nm bandwidth excitation filters at 485 nm (for YFP) and 540 nm (for RFP) and emission filters at 530 nm (for YFP) and 590 nm (for Cherry) readings. For FRET measurements, fluorescence was read in a FluoStar Optima Fluorimeter equipped with a high-energy xenon flash lamp, which allows the integration of the signals detected in the short-wavelength filter at 530 nm and the long-wavelength filter at 590 nm.

Net BRET and net FRET were defined as [(long-wavelength emission)/(short-wavelength emission)] − C_f_ where C_f_ corresponds to [(long-wavelength emission)/(short-wavelength emission)] for the donor construct expressed alone in the same experiment (Rluc for BRET and YFP for FRET). The GraphPad Prism software (San Diego, CA, USA) was used to fit the data. BRET and FRET are expressed as milli BRET units, mBU (net BRET × 1000) or milli FRET units, mFU (net FRET × 1000), respectively. The relative amount of BRET or FRET is given as a function of 1000 × the ratio between the fluorescence of the acceptor (YFP for BRET or Cherry for FRET) and the activity of the donor (Rluc for BRET or YFP for FRET).

For sequential resonance energy transfer (SRET) assays (see [12]), HEK-293T cells were transiently co-transfected with constant amounts of cDNA coding for the BRET donor (receptor-Rluc), the first acceptor (receptor-YFP) and increasing amounts of cDNA corresponding to the second acceptor (receptor-RFP). After 48 h, cells were detached using HBSS buffer with 0.1% glucose and cell suspension was adjusted to 20 µg/µL of protein using a Bradford assay kit (Bio-Rad, Munich, Germany) and bovine serum albumin (BSA) for standardization. Cells were treated with 1 µM methamphetamine or vehicle 1 h prior to each quantification. To quantify protein-RFP expression, fluorescence was read in a FluoStar Optima Fluorimeter (BMG Labtechnologies) using a 10-nm bandwidth excitation filter at 540 nm and an emission filter at 590 nm. Receptor fluorescence expression was determined as the fluorescence of the sample minus the fluorescence of cells expressing only receptor-Rluc and receptor-YFP. To quantify receptor-Rluc expression, luminescence readings were performed 10 min after 5 μM coelenterazine H addition using a Mithras LB 94 reader (Berthold Technologies). For SRET measurements, the cell suspension was treated with 5 μM coelenterazine H for 1 min and the SRET signal was determined using a Mithras LB 940 reader (485 nm) (short wavelength emission) and FluoStar Optima Fluorimeter (590 nm) (long wavelength emission). Net SRET was defined as [(long wavelength emission)/(short wavelength emission)] − Cf, where Cf corresponds to long wavelength emission/short wavelength emission for cells expressing only protein-Rluc and protein-YFP. SRET curves were fitted assuming a single phase by non-linear regression equation using the Graphpad Prism software (San Diego, CA, USA). SRET values are given as milli SRET units (mSU: 1000 × net SRET). The relative amount of SRET is given as a function of 1000 × the ratio between the fluorescence of the FRET acceptor (RFP) and the activity of the BRET donor (Rluc).

### 4.6. Cytosolic cAMP Determination

HEK-293T cells were transfected with the cDNA encoding for A_2A_R and/or for CB_1_R, and when indicated, with the siRNA for σ_1_R or for caln-1. Neuronal primary cultures were pretreated with 1 µM methamphetamine or vehicle for 2 h (acute treatment) or for one week (chronic treatment). Transfected HEK-293T cells were pretreated (20 min) with 1 µM methamphetamine or vehicle. Two hours before initiating the experiment, culture medium for transfected HEK-293T or primary neurons was replaced by serum-starved DMEM medium. Cells were detached, resuspended in DMEM medium containing 50 µM zardaverine (Tocris Bioscience, Bristol, UK) and plated (4000 cells/well) in white ProxiPlate 384-well microplates (PerkinElmer, Waltham, MA, USA). HEK-293T cells or neurons were treated with the corresponding antagonists (SCH 58261 for A_2A_R or SR 141716A for CB_1_R) or vehicle and stimulated (15 min) with the corresponding agonists (CGS 21680 for A_2A_R and/or ACEA for CB_1_R) or vehicle before adding 0.5 µM forskolin (15 min). Readings were performed after 1-h incubation at room temperature. Homogeneous time-resolved fluorescence energy transfer (HTRF) measurements were performed using the Lance Ultra cAMP kit (PerkinElmer, Waltham, MA, USA). Fluorescence at 665 nm was analyzed on a PHERAstar Flagship microplate reader equipped with an HTRF optical module (BMG Lab technologies, Offenburg, Germany). The effect of ligands was given in percentage with respect to the reference value.

### 4.7. Extracellular Signal-Regulated Kinase (ERK) and Protein Kinase B (Akt) Phosphorylation Assays

HEK-293T cells were transfected with the cDNA encoding for A_2A_R and/or for CB_1_R, and when indicated, with the siRNA of σ_1_R. Two to four hours before initiating the experiment, the culture medium was replaced by serum-starved DMEM medium. Cells were incubated at 37 °C with 1 µM methamphetamine (30 min) or vehicle, followed with the corresponding antagonist (SCH 58261 for A_2A_R or SR 141716A for CB_1_R) (20 min) and stimulated (7 min) with the corresponding agonists (CGS 21680 for A_2A_R and/or ACEA for CB_1_R). After the indicated incubation period, the reaction was stopped by placing cells onto ice. Then, cells were washed twice with cold PBS and lysed by the addition of ice-cold lysis buffer (50 mM Tris-HCl pH 7.4, 50 mM NaF, 150 mM NaCl, 45 mM ß-glycerolphosphate, 1% Triton X-100, 20 µM phenyl-arsine oxide, 0.4 mM NaVO_4_ and protease inhibitor mixture (MERK, St. Louis, MO, USA)). Cellular debris were removed by centrifugation at 13,000× *g* for 10 min at 4 °C, and protein was adjusted to 1 mg/mL by the bicinchoninic acid method (ThermoFisher Scientific, Waltham, MA, USA) using a commercial bovine serum albumin dilution (BSA) (ThermoFisher Scientific) for standardization. Finally, cells were denatured by placing them at 100 °C for 5 min. Akt and ERK1/2 phosphorylation were determined by western blot. Equivalent amounts of protein (20 µg) were subjected to electrophoresis (10% SDS-polyacrylamide gel) and transferred onto PVDF membranes (Immobilon-FL PVDF membrane, MERK, St. Louis, MO, USA) for 90 min. Then, the membranes were blocked for 1 h at room temperature (constant shaking) with Odyssey Blocking Buffer (LI-COR Biosciences, Lincoln, NE, USA) and labelled with a mixture of primary mouse anti-phospho-ERK 1/2 antibody (1:2000, MERK, Ref. M8159), primary rabbit anti-ERK 1/2 antibody (1:40,000, MERK, Ref. M5670), which recognizes both phosphorylated and non-phosphorylated ERK 1/2, and primary rabbit anti-phosphoAkt antibody (1:2500, Signalway Antibody, Baltimore, MA, USA, Ref. 11054) overnight at 4 °C with shaking. Then, the membranes were washed three times with PBS containing 0.05% tween and visualized by the addition of a mixture of IRDye 800 anti-mouse antibody (1:10,000, MERK, Ref. 926-32210) and IRDye 680 anti-rabbit antibody (1:10,000, MERK, Ref. 926-68071) for 2 h at room temperature. Membranes were washed 3 times with PBS-tween 0.05% and once with PBS and left to dry. Bands were analyzed using Odyssey infrared scanner (LI-COR Biosciences). Band densities were quantified using the scanner software, and the level of phosphorylated ERK 1/2 and Akt was normalized using the total ERK 1/2 protein band intensities. Results obtained are represented as the percent over basal (non-stimulated cells).

To determine the ERK 1/2 phosphorylation in striatal neuronal primary cultures, 50,000 neurons/well were plated in transparent 96-well microplates and kept in the incubator for 12 days. Neurons were pretreated with 1 µM methamphetamine for 2 h (acute treatment) or for one week (chronic treatment) or vehicle. Two to four hours before initiating the experiment, the medium was substituted by serum-starved DMEM medium. Then, neurons were pre-treated at room temperature for 15 min with the specific antagonist (SCH 58261 for A_2A_R or SR141716A for CB_1_R) or vehicle, and finally stimulated for 10 min with the specific agonists (CGS 21680 for A_2A_R and/or ACEA for CB_1_R). Neurons were then washed twice with cold PBS before the addition of 30 µL lysis buffer (20 min treatment in constant agitation). Subsequently, 10 µL of each supernatant were placed in white ProxiPlate 384-well microplates (PerkinElmer, Waltham, MA, USA) and the ERK 1/2 phosphorylation was determined using the AlphaScreen^®^SureFire^®^ kit (PerkinElmer) following the instructions of the supplier and using an EnSpire^®^ Multimode Plate Reader (PerkinElmer). The effect of ligands is given as percentage over the reference values.

### 4.8. Dynamic Mass Redistribution (DMR) Assays

Cell mass redistribution induced upon receptor activation was detected by illuminating the underside of a biosensor with a polychromatic light and measuring the changes in the wavelength of the reflected monochromatic light, which was a sensitive function of the index of refraction. The magnitude of this wavelength shift (in picometers) was directly proportional to the amount of dynamic mass redistribution (DMR). HEK-293T cells co-transfected with the cDNA coding for A_2A_R and for CB_1_R were seeded in 384-well sensor microplates (Corning^®^ Epic^®^ 384-well Cell Assay Microplate) (MERK, St. Louis, MO, USA) to obtain a 70–80% confluent monolayer constituted by approximately 10,000 cells per well. Before the assay, cells were washed twice with assay buffer (HBSS with 20 mM HEPES pH 7.15 containing 0.1% DMSO) and maintained for 2 h at 24 °C in the same buffer (30 µL/well). Then, the sensor plate was scanned, and a baseline optical signature was recorded for 10 min before adding 10 µL of the specific antagonists (SCH 58261 for A_2A_R or SR141716A for CB_1_R) in the presence or in the absence of 1 µM methamphetamine and recorded for 30 min, followed by the addition of 10 µL of the specific agonists (CGS 21680 for A_2A_R and/or ACEA for CB_1_R) and monitoring for another 60 min. All of the tested compounds were dissolved in the assay buffer. The cell signaling signature was determined using an EnSpire^®^ Multimode Plate Reader (PerkinElmer, Waltham, MA, USA) by a label-free technology. Results were analyzed using the EnSpire Workstation Software v 4.10 (PerkinElmer).

### 4.9. Determination of Cytoplasmic Calcium Ion Levels

HEK-293T cells were co-transfected with the cDNAs coding for A_2A_R, CB_1_R and GCaMP6 calcium sensor. At 48 h post-transfection, cells were detached using Mg^2+^ Locke’s buffer (154 mM NaCl, 5.6 mM KCl, 3.6 mM NaHCO_3_, 2.3 mM CaCl_2_, 5.6 mM glucose and 5 mM HEPES, pH 7.4) supplemented with 10 µM glycine, and the cell suspension was adjusted to 40 µg/µL of protein using a Bradford assay kit (Bio-Rad, Munich, Germany) and BSA for standardization. Cells were plated in 96-well black with clear bottom microplates (Porvair) and pretreated with 1 µM methamphetamine or vehicle for 1 h before treatment with the specific antagonists (SCH 58261 for A_2A_R or SR 141716A for CB_1_R) for 10 min, followed by the addition of the specific receptor agonists (CGS 21680 for A_2A_R and/or ACEA for CB_1_R) or 1 µM ionomycin a few seconds before readings. Fluorescence emission intensity of the GCaMP6 was recorded at 515 nm upon excitation at 488 nm on the EnSpire^®^ Multimode Plate Reader (PerkinElmer, Waltham, MA, USA) for 150 s every 5 s.

### 4.10. ß-Arrestin 2 Recruitment

HEK-293T cells were co-transfected with the cDNA coding for A_2A_-YFP, CB_1_R and ß-arrestin 2-Rluc. At 48 h post-transection, cells were detached using 0.1% glucose HBSS buffer and the cell suspension was adjusted to 30 µg/µL of protein using a Bradford assay kit (Bio-Rad, Munich, Germany) and BSA for standardization. Cells were plated in 96-well black with clear bottom plates (Porvair) and pretreated with 1 µM methamphetamine or vehicle for 30 min before treatment with the specific antagonists (SCH 58261 for A_2A_R or SR 141716A for CB_1_R) for 10 min. Coelenterazine H (5 µM) (PJK GMBH, Kleinblittersdorf, Germany) was added before stimulation with the specific agonists (CGS 21680 for A_2A_R and/or ACEA for CB_1_R) for 2 min. Then, BRET between ß-arrestin 2-Rluc and A_2A_R-YFP was determined as described above (see Resonance Energy Transfer Assays).

### 4.11. Proximity Ligation Assays (PLA)

Striatal neuronal primary cultures grown on glass coverslips were pretreated with 1 µM methamphetamine for 2 h (acute treatment) or for one week (chronic treatment) or vehicle. In brief, neurons were fixed in 4% paraformaldehyde for 15 min, washed with PBS containing 20 mM glycine to quench the aldehyde groups, and permeabilized with the same buffer containing 0.05% Triton X-100 for 5 min. After 1 h incubation at 37 °C with blocking solution, the neurons were treated with specific antibodies against A_2A_, CB_1_ or σ_1_ receptors. The following combinations of antibodies were used: mouse monoclonal anti-A_2A_R (1/100, Santa Cruz Biotechnology, Dallas, Paisley, UK, Ref. 32261) and rabbit polyclonal anti-CB_1_R (1/100; ThermoFisher Scientific, Waltham, MA, USA, Ref. PA1-745), rabbit polyclonal anti-A_2A_R (1/100, ThermoFisher Scientific, Ref. PA1-042) and mouse monoclonal anti-σ_1_R (1/100, Santa Cruz Biotechnology, Ref. 137075) or rabbit polyclonal anti-CB_1_R (1/100, ThermoFisher Cientific) and mouse monoclonal anti-σ_1_R (1/100, Santa Cruz Biotechnology). Cells were processed using the PLA probes detecting mouse and rabbit antibodies (1/100, Duolink II PLA probe anti-rabbit plus and Duolink II PLA probe anti-mouse, MERK, St. Louis, MO, USA) in the presence of Hoechst (1/100, MERK). Preparations were mounted using 30% Mowiol (Calbiochem, San Diego, CA, USA). Negative controls were performed by omitting one of the primary antibodies. Images were obtained in a Leica SP2 confocal microscope (Leica Microsystems, Mannheim, Germany) equipped with apochromatic 63X oil-immersion objectives (N.A. 1.4), and 405 and 561 nm laser lines. For each field of view, a stack of two channels (one per staining) and 3 to 4 Z stacks with a step size of 1 µm were acquired. The number of cells containing one or more red spots versus total cells (blue nucleus) and, in spot-containing cells, the ratio r (number of red spots/cell), were determined using the Duolink Image tool software (MERK).

### 4.12. Statistical Analysis

Graph data are the mean ± SEM (*n* = 6 at least). GraphPad Prism software version 8 (San Diego, CA, USA) was used for the data fitting and statistical analysis. A one-way or two-way ANOVA followed by the post-hoc Bonferroni’s test were used when comparing multiple values. Significant differences were considered when the *p* value was <0.05.

## Data Availability

The data presented in this study are available on request from the corresponding author. The data are not publicly available due to privacy restrictions.

## Abbreviations

A_2A_R	Adenosine A_2A_ receptor
BRET	Bioluminescence Resonance Energy Transfer
BSA	Bovine Serum Albumin
CB_1_R	Cannabinoid CB_1_ receptor
CB_2_R	Cannabinoid CB_2_ receptor
CGS	CGS 21680
CNS	Central nervous system
D_1_R	Dopamine D_1_ receptor
DMEM	Dulbeco’s Modified Eagle’s Medium
DMR	Dynamic Mass Redistribution
GPCR	G Protein-Coupled Receptor
FBS	Fetal Bovine Serum
FK	Forskolin
FRET	Fluorescence Resonance Energy Transfer
HTRF	Homogeneous time-resolved fluorescence energy transfer
Met	Methamphetamine
MAPK	Mitogen activated protein kinase
PBS	Phosphate-buffered saline
PLA	Proximity Ligation Assay
SCH	SCH 58261
SR	SR 141716A
SRET	Sequential Resonance Energy Transfer
∆^9^-THC	∆^9^-Tetrahydrocannabinol
σ_1_R	Sigma 1 receptor
